# Patient Smartphone Ownership and Interest in Mobile Apps to Monitor Symptoms of Mental Health Conditions: A Survey in Four Geographically Distinct Psychiatric Clinics

**DOI:** 10.2196/mental.4004

**Published:** 2014-12-23

**Authors:** John Torous, Steven Richard Chan, Shih Yee-Marie Tan, Jacob Behrens, Ian Mathew, Erich J Conrad, Ladson Hinton, Peter Yellowlees, Matcheri Keshavan

**Affiliations:** ^1^ Harvard Longwood Psychiatry Residency Training Program Boston, MA United States; ^2^ Beth Israel Deaconess Medical Center Department of Psychiatry Harvard Medical School Boston, MA United States; ^3^ General Psychiatry Residency Training Program UC Davis School of Medicine Sacramento, CA United States; ^4^ Department of Psychiatry and Behavioral Sciences UC Davis School of Medicine Sacramento, CA United States; ^5^ Louisiana State University-Ochsner Psychiatry Residency Program Louisiana State University Health Sciences Center Louisiana State University New Orleans, LA United States; ^6^ Department of Psychiatry Louisiana State University Health Sciences Center New Orleans, LA United States; ^7^ Department of Psychiatry University of Wisconsin School of Medicine and Public Health Madison, WI United States

**Keywords:** psychiatry, mobile health, smartphone

## Abstract

**Background:**

Despite growing interest in mobile mental health and utilization of smartphone technology to monitor psychiatric symptoms, there remains a lack of knowledge both regarding patient ownership of smartphones and their interest in using such to monitor their mental health.

**Objective:**

To provide data on psychiatric outpatients’ prevalence of smartphone ownership and interest in using their smartphones to run applications to monitor their mental health.

**Methods:**

We surveyed 320 psychiatric outpatients from four clinics around the United States in order to capture a geographically and socioeconomically diverse patient population. These comprised a state clinic in Massachusetts (n=108), a county clinic in California (n=56), a hybrid public and private clinic in Louisiana (n=50), and a private/university clinic in Wisconsin (n=106).

**Results:**

Smartphone ownership and interest in utilizing such to monitor mental health varied by both clinic type and age with overall ownership of 62.5% (200/320), which is slightly higher than the average United States’ rate of ownership of 58% in January 2014. Overall patient interest in utilizing smartphones to monitor symptoms was 70.6% (226/320).

**Conclusions:**

These results suggest that psychiatric outpatients are interested in using their smartphones to monitor their mental health and own the smartphones capable of running mental healthcare related mobile applications.

## Introduction

The utility of mobile mental health has become a topic of increasing interest to psychiatric researchers, industry, and the public. Furthermore, the role of smartphones for clinical monitoring and care of psychiatric patients is receiving significant attention. Recent research has investigated the feasibility and potential of smartphone applications in the care of patients suffering from major depressive disorder [1], bipolar disorder [2], anxiety disorders [3], substance abuse disorders, [4,5] and psychotic disorders [6,7]. However, one fundamental question remains largely unanswered despite such research advances: do patients with psychiatric conditions actually own smartphones and, if so, are they interested in using their personal devices to run clinical monitoring or treatment applications?

The only previous study addressing this question suggested that psychiatric outpatients at a university outpatient clinic in Boston owned smartphones at a rate of 72% [8], greater than the current United States average ownership rate of 58% in January 2014 [9]. In this expanded study, we sought to perform the same survey and protocol as in the Boston-based study in four new psychiatric clinics located across the country in each of the four distinct US census districts.

We aimed to capture opinions from a broad range of psychiatric patients by studying different clinic settings. Patients with serious and chronic mental illness are an important population to include, given the unique challenges in caring for this population. Patients with private insurance may also be very ill, but have different resources available to them and thus represent another important population to study. Finally, those patients with more variable resources are also important to consider, given that many psychiatric patients may not qualify for state level of care but also not have private insurance. County clinics often serve these patients and feature sliding scale payments to meet patients’ ability to pay. Clinic type has also been used as a proxy for patient socioeconomic status [10]; thus, including multiple clinic sites helps ensure that a diverse population is captured.

We hypothesized that those patients with serious and chronic mental illness would have a lower prevalence of ownership than those patients in hybrid or county clinics, and that patients at a private insurance clinic would have the highest prevalence of ownership. Based on prior research, we expected that all patient groups would demonstrate high levels of interest in running smartphone applications to monitor their mental health on their personal devices. Finally, we hypothesized that younger patients would have both higher ownership and interest in utilizing their smartphones for monitoring than older patients.

## Methods

A total of four study sites conducted the survey. The first study site included a state-run outpatient psychiatric clinic with a partial hospital program, and a 40-bed transitional residential program for those with largely serious and chronic mental illness in Boston, Massachusetts. The second site was a system of two county-run community outpatient psychiatry clinics that serves largely independently functioning patients in Sacramento, California. The third site was a hybrid clinic that treats a majority of patients with public insurance but also sees roughly one third of patients with private insurance in New Orleans, Louisiana. The fourth site was a university outpatient psychiatry clinic that serves a largely privately insured population in Madison, Wisconsin.

Identical paper-and-pencil surveys assessing patients’ smartphone ownership and interest in using personal smartphones phones to monitor mental health were distributed to each of the four study clinics.  A copy of the survey questions are displayed in Textbox 1. The surveys were made available to all patients in clinic who voluntarily filled them out before or during appointments, and submitted completed forms to the clinic. Surveys, along with handouts explaining the purpose, mental health focus, and voluntary nature of the study, were offered and provided to patients by clinic staff at all sites while patients were waiting for appointments. All surveys were completed in the clinic setting. All clinic patients were eligible. The survey was made available for 4 weeks at each study site, with all sites completing the data collection in either July or August 2014.

Patients received no compensation or incentives to complete surveys, and study personnel collected completed surveys at least weekly. Results were entered into password-protected spreadsheet software, and all analyses and graphs were completed in the R programming language. We used Pearson chi-squared goodness of fit tests to compare distributions of groups. The Institutional Review Boards at each of the respective study sites approved the study, and a waiver of informed consent was obtained for each site.

**Questions from the paper survey used for the study.**

1) Do you currently have daily access to the Internet? ☐Yes or ☐No

2) Do you currently own a mobile phone? ☐Yes or ☐No

3) Can your phone receive and send text messages? ☐Yes or ☐No

4) Can your phone browse the Internet? ☐Yes or ☐No

5) Can your phone download applications or “apps”? ☐Yes or ☐No

6) Does your phone have GPS built in? ☐Yes or ☐No

7) Do you own smartphone? ☐Yes or ☐No

8) What is the brand and type of your mobile phone (eg Apple iPhone).

9) How many applications or “apps” do you have on your phone?

10) How many applications or “apps” do you put on your phone each month?

11) How many health care related applications or “apps” to you have on your phone?

12) In the last six months, have you used your smartphone to access general health care information? ☐Yes or ☐No

13) In the last six months, have you used your phone to access your personal health care information such as for example test results or to schedule appointments? ☐Yes or ☐No

14) Would you want to be able to access general information related to your health via your smartphone? ☐Yes or ☐No

15) Would you want to receive text messages on your phone related to your health from your doctor’s office? ☐Yes or ☐No

16) Would you want to use your phone to help track your medical condition via an application or “app” on your smartphone? ☐Yes or ☐No

17) Would you download an application or “app” to your phone to help monitor your health condition? ☐Yes or ☐No

18) Would you be willing to use an application or “app” on your phone on a daily basis to help monitor your health condition? ☐Yes or ☐No

## Results

A total of 320 patients completed the survey at all study sites. This comprised of 106 (33.1%; 50/106, 47.2% female) patients at the state clinic in Boston, 50 (15.6%; 28/50, 56% female) at the hybrid clinic in New Orleans, 56 (18%; 36/56, 64% female) at the county clinics in Sacramento, and 108 (33.8%; 51/108, 47.2% female) at the university clinic in Madison. In total, 52% of total respondents were female. The mean age of respondents was 43.7 years. The mean age at the state clinic in Boston was 43.9 years, 39.6 years at the Sacramento county clinic, 44.7 years at the New Orleans hybrid clinic, and 36.2 years at the private clinic in Madison.

As the survey was not monitored, it was difficult to know exactly what percentage of patients chose to complete it. Based on estimates of patient volume, we believe that roughly 10% of patients at each clinic site took the survey. Although we were not able to physically examine patients’ phones, of the 184 patients who answered question 7—indicating that they owned a smartphone—all 184 also responded affirmatively to questions 3 through 6, indicating that features of their phones included the ability to send text messages, browse the Internet, download apps, and track location using GPS.

The total average for smartphone ownership, question 7, was 62.5% (200/320) for all study sites. The overall willingness to use a smartphone app to monitor their mental health was 70.6% (question 16; 226/320).

**Figure 1:**
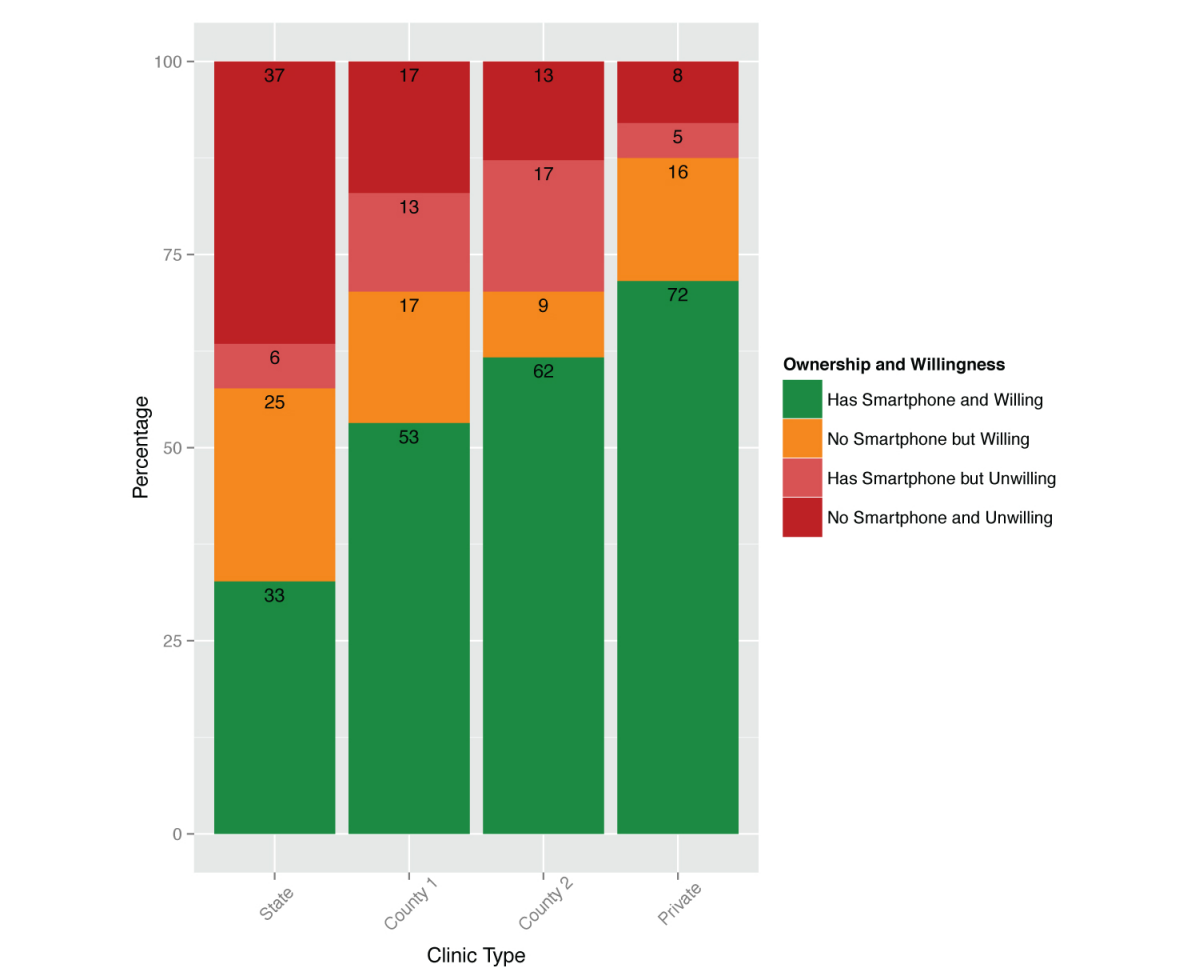
Percent ownership of smartphones (question 7) and interest in using a smartphone to monitor mental health conditions (question 16) by clinic.

We analyzed results for each of the 4 sites. At the state-run clinic, 38.7% (41/106) of patients reported owning a smartphone and 57.5% (61/106) were willing to use a smartphone to monitor their mental health. At the hybrid clinic, the smartphone ownership was 66% (33/50) and willingness to use was 70% (35/50). At the county clinic system, smartphone ownership was 79% (43/56) and willingness to use was 71% (40/56). Finally, ownership at the university clinic was 76.9% (83/108), and willingness to use was 88.0% (95/108). Details of the results by study site are displayed in Figure 1.

We also analyzed results by age groupings in a similar fashion to prior studies [8]. To further study age effects, we categorized patients into age buckets of those less than 30 (n=85), between age 30 and 45 (n=120), between age 45 and 60 (n=72), and those older than 60 (n=31). Twelve patients did not include their age and were not included in this analysis. Of note, there was a significant difference between these age groupings, with a *P* value of .002 (χ^2^_9_=26.09).

**Figure 2:**
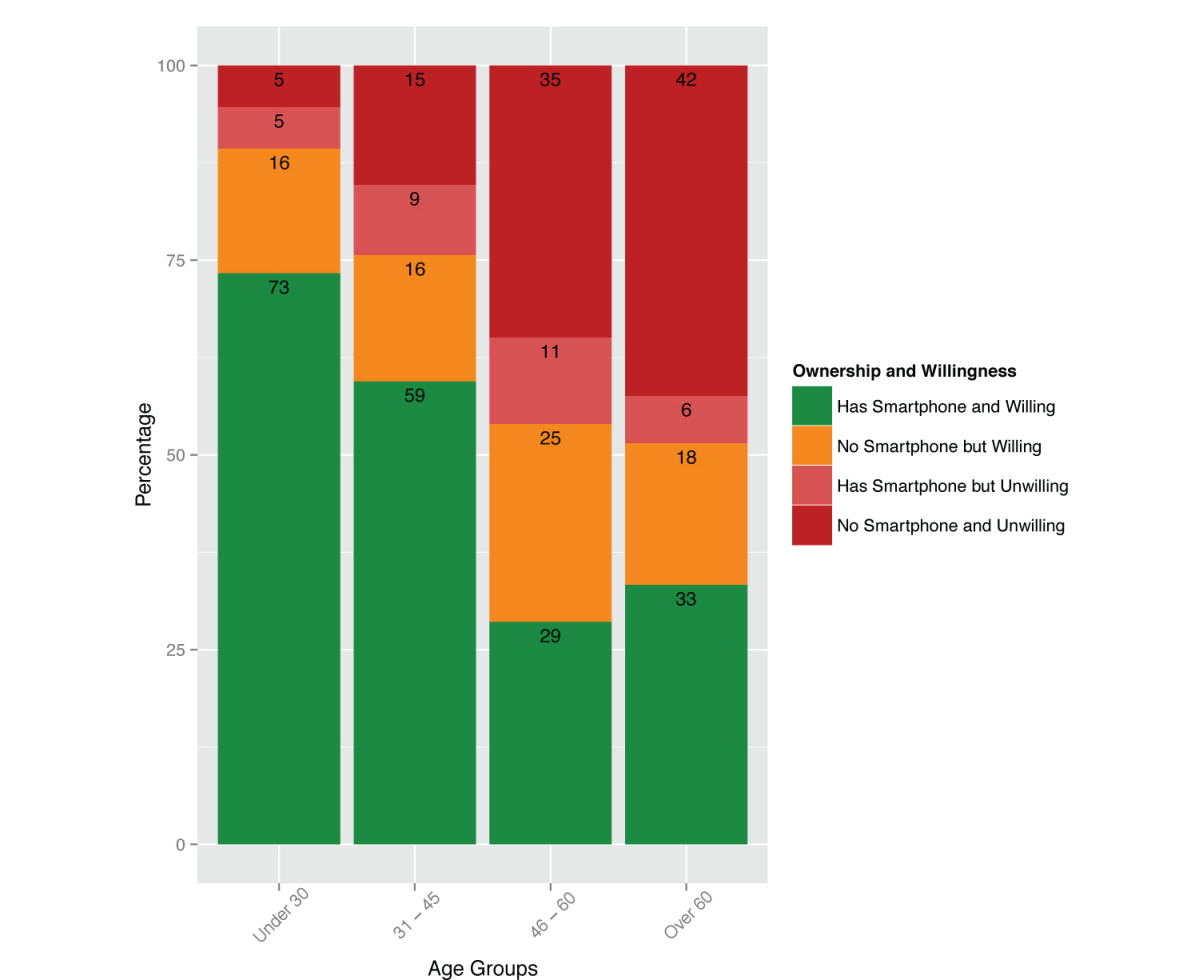
Percent ownership of smartphones (question 7) and interest in using a smartphone to monitor mental health conditions (question 16) by age.

For patients under thirty years of age, percent ownership was 78% (66/85) and willingness to use to monitor mental health was 89% (76/85). For patients between ages 30 and 45, ownership was 68.3% (82/120) and willingness to use was 75.0% (90/120). For patients between ages 45 and 60, ownership was 40% (29/72) and willing to use was 54% (39/72). Finally, for patients over 60, ownership was 39% (12/31) and interest was 51% (16/31). Details of results by age are shown in Figure 2. Responses to other survey questions, stratified by age, are shown in Table 1.

**Table 1:** Response to other survey questions, stratified by age.

Question	Under 30 years (n=85)	31-45 years (n=120)	46-60 years (n=72)	Over 60 years (n=31)	Average Response (n=308)
	n (%)	n (%)	n (%)	n (%)	n (%)
Q1: Daily Access to Internet?	85 (88.2)	96 (80.0)	53 (73.6)	22 (71.0)	246 (79.9)
Q2: Owning any Mobile Phone? (not necessarily a Smartphone)	76 (89.4)	109 (90.8)	58 (80.6)	23 (74.2)	266 (86.4)
Q12: Used a Smartphone in Last Six Months to Access General Health Information?	57 (67.1)	68 (56.7)	18 (25.0)	9 (29.0)	152 (49.4)
Q13: Used a Smartphone in Last Six Months to Access Personal Health Information?	39 (45.9)	33 (27.5)	12 (16.7)	5 (16.1)	89 (28.9)
Q15: Want to Receive Text Messages Related to Your Health?	67 (78.8)	85 (70.8)	39 (54.2)	18 (58.1)	209 (67.9)
Q18: Would you use an App to Monitor Your Health on a Daily Basis?	53 (62.4)	73 (60.8)	32 (44.4)	16 (51.6)	174 (56.5)

## Discussion

### Principal Findings

Our results confirmed the hypothesis that psychiatric outpatients’ ownership and interest in utilizing smartphones varied by clinic setting and age. Across all sites, average smartphone ownership was 62.5%, which is similar to the United States national average of 58% as of January 2014 [9]. Our results also suggest that, overall, psychiatric outpatients are favorable to the idea of using their own smartphones to run applications to monitor their mental health, with 70.6% favoring this assessment modality. Even in the state-run clinic, which had the lowest rates of both patient ownership and interest, the results were positive: 39% reported owning a smartphone, and 58% expressed a willingness to use a smartphone to monitor their condition. However, contrary to our first hypothesis, patients in the county clinic system, and not the private clinic, had the highest average rate of smartphone ownership, although the results only differed by 1% between these two clinics.

Such results are important as they suggest that implementing smartphone application based clinical monitoring and treatment protocols may involve fewer patient obstacles and less resistance than commonly thought. The results are also important as they underscore the potential to implement digital interventions or monitoring at a lower implementation cost, given that patients are willing to use their own personal smartphones. Mirroring national trends for smartphone ownership, our data also suggests that the youngest generation, represented by those less than 30 years of age in our study, had the highest rates of ownership and willingness to engage in this modality.

Our results also suggest areas of opportunity and growth for mobile mental health. As smartphones become cheaper in price and progress towards ubiquity, it is likely that those patients in our survey who indicated that they do not currently own a smartphone but would be willing to use such to monitor their health will soon have that opportunity. In our study, the number of patients who owned a smartphone but were unwilling to use this modality to monitor their health was relatively small. This suggests that while some patients will remain resistant to mobile mental health technologies, it is likely that overall patient interest and engagement in such will continue to grow.

Taken together, these results suggest that smartphone monitoring and intervention studies targeting younger patients with private insurance may be easier to implement than in other patient environments, such as with elderly patients in state-based clinics. Thus, when interpreting the results of feasibility studies for mobile mental health, it may be important to understand that both age and socioeconomic demographics are likely independent variables that must also be taken into account.

Looking at patient connectivity beyond smartphones, our data suggests that, on average, 80% of patients have access to the Internet and that 86% own a mobile phone. Thus, to reach those patients currently without smartphones or Internet access, text messaging apps remain a viable solution. Interestingly, while 72% of patients in our study were willing to use an app to monitor their mental health, only 68% wanted to receive text messages related to their health. While we did not collect data on why patients may prefer apps to text messaging, the dynamic interactivity and visual format of apps may be easier to use and respond to than text messaging, comprised of static text with strict character limits.

### Comparison With Other Studies

Our results are in line with and supported by previous research. A similar study of psychiatric outpatients at a different university clinic that accepts largely private insurance reported 72% smartphone ownership and 68% willingness to use such to monitor their mental health [8], while in this study, the university clinic patients had 77% ownership and 88% willingness to use such apps. A study of schizophrenia patients with chronic illness noted that 28% owned a smartphone [7], which is slightly lower than our rate of 39% ownership in the state clinic that treats largely those with serious mental illness. A recent study of 189 psychiatric outpatients in an inner-city community psychiatric clinic reported that 85.7% of patients in that study owned a mobile phone [11]; similarly, in our study, the average rate of mobile phone ownership was 86%.

### Limitations

Our study has several limitations. First, our results are based on survey data and responses about using an app are hypothetical and not verified in practice. While 70.6% of patients reported interest in using an app to monitor their mental health, only 49% indicated they had used a smartphone to look up general health information in the six months preceding the survey, and only 29% had used such to look up personal health information. Second, we did not collect data on those who chose not to partake in the survey, and this may have skewed our results to be more positive. Third, we did not collect data related to individual diagnoses, so we are not able to further analyze and compare between patients with specific disorders such as, for example, patients with depression versus anxiety versus bipolar disorder. Fourth, we did not control for potential differences in smartphone ownership rates in each community where the study clinics were located.

### Conclusions

In conclusion, our results suggest that psychiatric outpatients may own smartphones at near the national average and that overall patient interest in using smartphones to monitor their mental health is high. Our results varied based on age and clinic type, suggesting that both are important factors to consider when designing a study or implementing a treatment intervention. Many psychiatric outpatients have smartphones and are interested in using them regarding their mental health. The next challenge is whether psychiatry can meet that interest with clinically valid and effective apps.
